# Evaluation of ergonomic factors and postures that cause muscle pains in dentistry students’ bodies

**DOI:** 10.4317/jced.51909

**Published:** 2015-07-01

**Authors:** Masoumeh Shirzaei, Ramazan Mirzaei, Ali Khaje-Alizade, Mahdi Mohammadi

**Affiliations:** 1 Assistant Professor of mouth diseases and member of the research center of Dentistry Faculty of Zahedan, Iran; 2 Associate Professor of Occupational Health, Health Promotion Research Center, Zahedan University of Medical Sciences, Zahedan, Iran; 3 Dental Practitioner, Zahedan University of Medical Sciences, Zahedan, Iran; 4 Associate Professor of Biostatistics, Health Promotion Research Center, Zahedan University of Medical Sciences, Zahedan, Iran

## Abstract

**Background:**

Work-related musculoskeletal disorders commonly experienced by dental professionals are one of the main occupational health problem affecting their health and well-being.This study was conducted to evaluate ergonomic factors and profession-related postures and also investigate relationship between demographic factors and work condition with pain in dental students.

**Material and Methods:**

60 freshman and sophomore dentistry students were randomly chosen as the subjects of control group, and 60 of 5th and 6th-year students were selected as the members of exposure group. Data related to the subjects such as sex, doing exercise, severity of musculoskeletal pain were obtained through questionnaire. Students’ postures were directly observed while treating patients and they were scored by REBA method. Data were analyzed by SPSS software using Man-Whitney, Kruskal-Wallis, Spearman and Kendall correlation tests.

**Results:**

80.8% of the subjects were not aware of the correct ergonomic postures for dental procedures. Severity of musculoskeletal pain in the exposure group (15.9± 4.2) was significantly higher than the control group (10.5 ±3.2), (*p* <0.001). Risk of the most subjects (84%) was at the medium level. Students who were more involved in clinical activities experienced more muscular pains.

**Conclusions:**

The musculoskeletal disorders are probable prolonged in working hours in static positions, incorrect work postures, implying more force and even tools and instruments. Therefore, students who are aware of ergonomic principals of their own profession would be able to maintain their health through activities and lifelong.

** Key words:**Posture, dentistry, students, musculoskeletal pain.

## Introduction

Ergonomy is a set of scientific principals correlating to understanding of the interactions between humans and other elements of a system. Work strain and occupational damages to humans, and preventing profession-related disorders could be evaluated by ergonomic principals.

Work- related musculoskeletal disorders (WMSDs) is a major problem in modern societies (1,2) and refer to every kind of tissue damages to the musculoskeletal system and to the nerves; dentists are more likely to have musculoskeletal disorders due to their job. Dentistry, especially general dentistry, is one of the dangerous and stressful professions. Improper prolonged repetitive working habits in addition to observational requirements of this field as well as recurrent movements of the upper body/upper limb are the reasons of these kinds of problems in this profession (3).

Long-term working periods and short periods of rest and repetitive movements of organs apply statically constant high pressure on some parts of the body resulting in pain, spasm, tingling, and stiffness of joints and so on and so forth. Thus, the routine life of a dentist is affected in long time and might cause his/her early resignation.

During recent years, work environment of dentists and dentistry students have been studied in different parts of the world. Some researchers are convinced that the frequency of disorders has increased by changing standing sitting position. These researchers state that, this change of position while working might not be the only effective factor; other factors such a work environment, mental pressure, non-stopping work periods, and the visual status of dentists are effective as well (4).

Caballero *et al.* did a study on 83 dentistry students of University of Colombia about ergonomic factors. 80% of them suffered from muscular pains due to clinical work, 55% thought that using hand tools without non-essential movements is useful, and only 13% of the students were exercising during their education years. In general, doing surgery and periodontics increased job stress (5).

Rising *et al.* determined the distribution and severity of musculoskeletal pains in 270 dentistry students of California by body map. Neck and shoulder pain have been reported for women and waist pains/ backaches for men. The frequency and pain period of higher educated students was more than others, and the severity of pain in women was more than men (6).

Moreover, Rafeemanesh *et al.* evaluated the ergonomic conditions of 65 dentists (working in the health centers of Mashhad, Iran) using rapid entire body assessment method. The highest and the lowest REBA scores were 11 and 4 respectively. The prevalence of musculoskeletal disorders for neck, shoulders, upper back, lower back and wrists was 75.9%, 58.6%, 56.9%, 48.3% and 44.8% respectively (7).

Different kinds of ergonomic factors like body position while treating patients have a crucial role in causing musculoskeletal disorders. For instance, dentists need to be more accurate for root canal treatment which makes them to deviate from the normal position and to be in a high level of stress. This study was conducted with the aim of evaluating ergonomic factors and profession-related postures and also relationship between demographic factors and work condition with pain in dental students of Zahedan, Iran.

## Material and Methods

This cross-sectional study was conducted in dental school of Zahedan University of Medical Science located in South-East of Iran in 2013. Dental students were divided into two groups: First and second year students with no clinical practice (control group) and fifth and sixth year students with clinical practice (exposure group). A sample of 60 students (30 male and 30 female) was randomly selected from each of abovementioned groups. After taking permission, a questionnaire including sex, education year, severity of musculoskeletal pains and doing stretching exercises were filled up by dentistry students in the classrooms. Subjects with any diagnosed musculoskeletal diseases history (e.g., Arteritis, chronic backache, musculoskeletal deformity) were excluded from the study.

**Figure 1:**
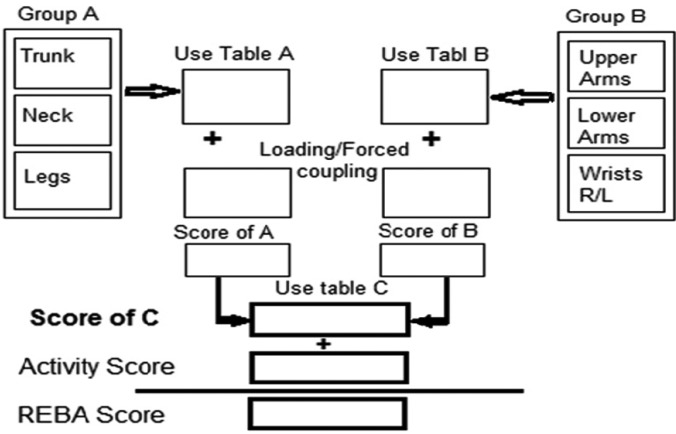
REBA Score sheet.

Physical posture and the potentiality of profession-related musculoskeletal disorders in the whole body were determined through REBA method (8-11). REBA method is a rapid and easy observational postural analysis tool for whole body activities which evaluate the ergonomics risk factor by observing the posture of employees while they are working at their workstation directly. Each of body organ postures is given a score by which posture scores are determined based on the figure 1 diagram. Accordingly, A and B scores are taken from the standard tables and loading/forced coupling scores are determined based on the load of the instrument to the hands while working (if load <11 lbs. =0, 11-22lbs=1and so on). Then loading score is added to scores A and B and finally REBA score are obtained and the risk level is determined based on  Table 1 for corrective action decision.

**Table 1:**
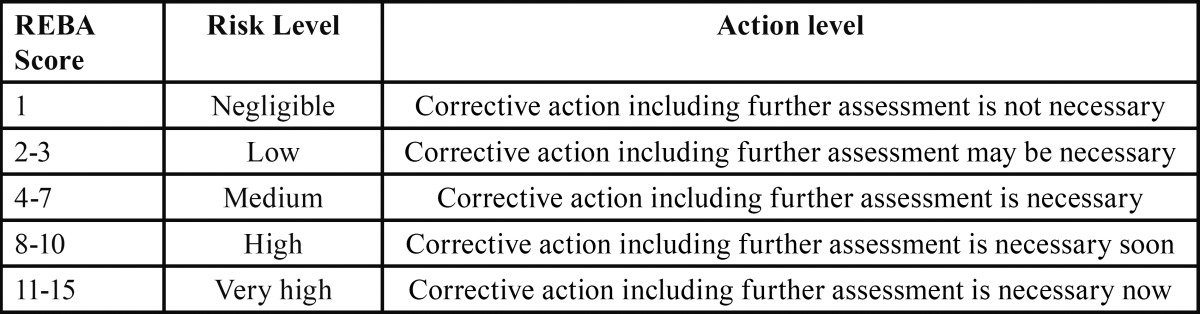
REBA Decision.

For ascertaining the intensity of musculoskeletal pain, the student ranked the acuteness of pain in the mentioned organs from 1 to 5 and then all scores from 9 organs were added up. Therefore, the severity of musculoskeletal pains was ranked from 9 (no pain) to 45 (the most amount of pain). Afterwards, each subject was monitored in the treatment center for determining his/her body position while working, and their potentiality of musculoskeletal disorders was scored by REBA. Scoring was done on a determined body positions while the students were treating the patients (the student did not notice the scoring stage.). Next, the scores were written in REBA score sheet (Fig. 1), and the final scores were determined.

After coding the REBA data and Questionnaire information, they were entered to SPSS software (version 18) and were analyzed by Mann-Whitney, Kruskal-Wallis, Spearman and Kendall correlation tests.

## Results

The results demonstrate that 80.8% of the exposure group students were not aware of correct ergonomic postures for optimizing clinical activities. 58.3% of students in this group adjusted their seat for adopting a correct form of position before work. The mean of the musculoskeletal pains of the exposure group (15.9±4.2) was significantly higher than that of the control group (10.5± 2.3); (*p*<0.001).

In  Table 2, the average severity of musculoskeletal pains of the exposure and control groups has been classified based on their gender. The average pain in female students was more than male students; however, there was no significant relationship between gender and pain (*p*>0.05). In each gender, the average intensity of musculoskeletal pains in the exposure group was significantly higher than that in the control group (*p*<0.001).

**Table 2:**
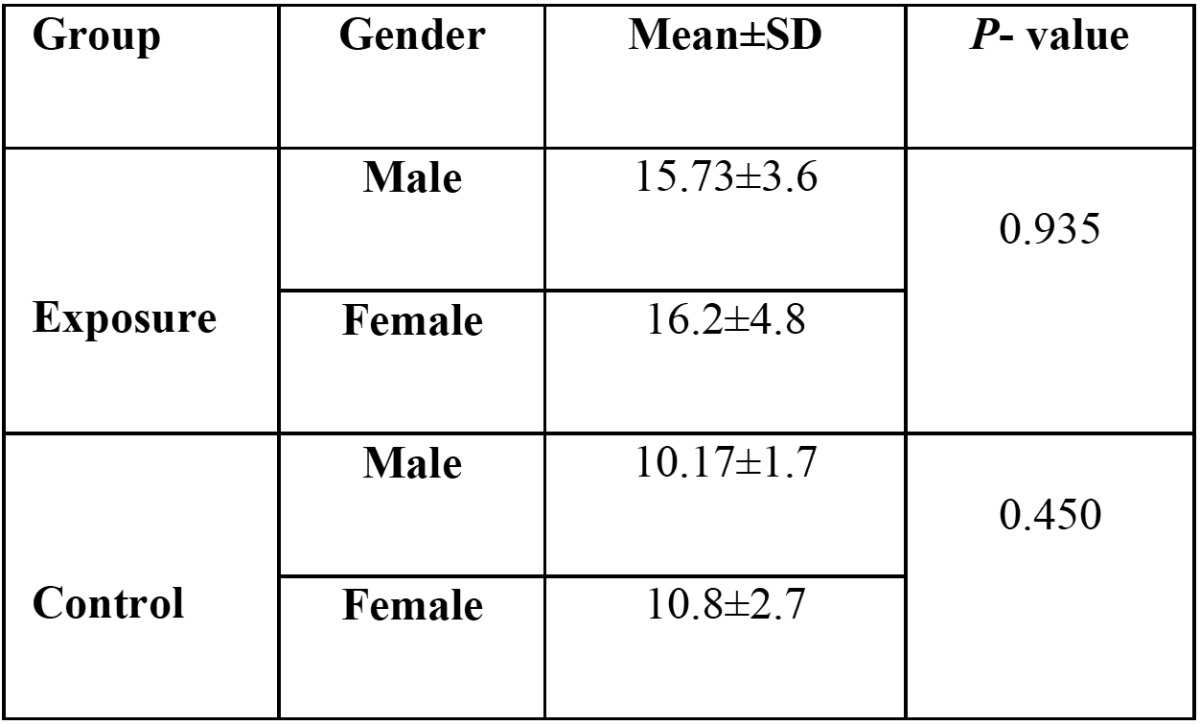
Average intensity of musculoskeletal pains of the control and exposure groups with regard to gender (SD= Standard deviation).

The average severity of musculoskeletal pains with regard to clinical activities in the exposure group has been revealed in  Table 3. The highest and lowest average of pain was in the surgery (16.6 ± 4.3) and reconstructive (15.3 ± 5.1) departments respectively. Nevertheless, there was no significant difference among various types of clinical activities (*p*> 0.05).

**Table 3:**
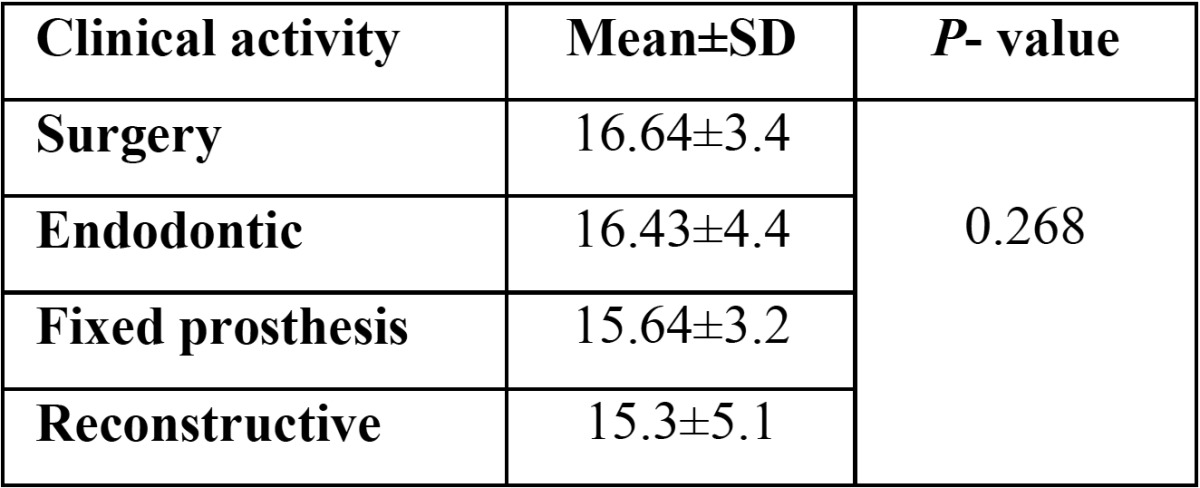
Average intensity of musculoskeletal pains in the exposure group’s students with regard to clinical activities.

In the exposure group, the average acuteness of musculoskeletal pains was 13.2 ± 2.1 for subjects aware of ergonomic postures compared to 22.75 ± 3.5 for unaware students (*P*= 0.046).

In each group, the average severity of musculoskeletal pains was not significantly related to the duration of computer use in a day (*P*>0.05).

According to  Table 4, the average severity of musculoskeletal pains decreased significantly by doing exercise from 20.4±3.5 to 12.8±4.1 in the exposure group (*P*=0.039) and from 15.7±2.8 to 10.2±1.8 in the control group (*P*=0.045).

**Table 4:**
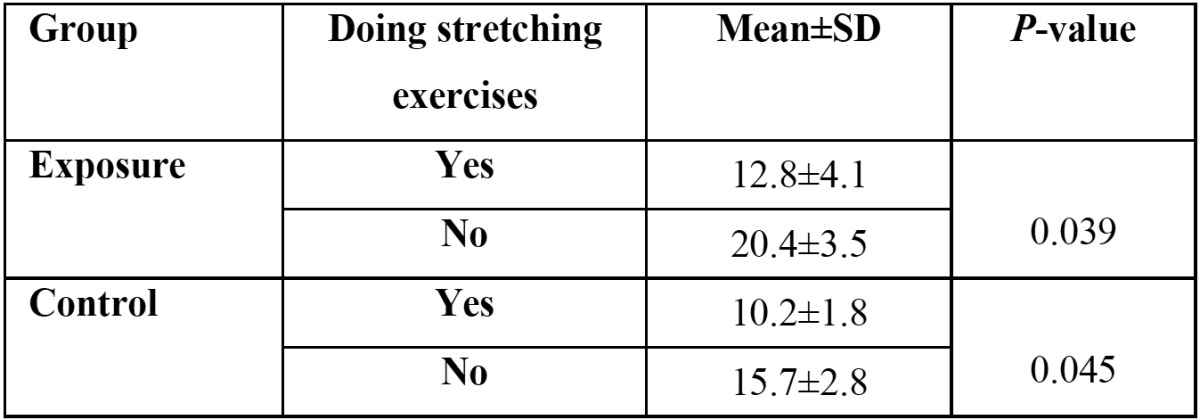
Average intensity of musculoskeletal pains in the students of the control and exposure groups based on doing stretching exercises after treating patients.

**Figure 2:**
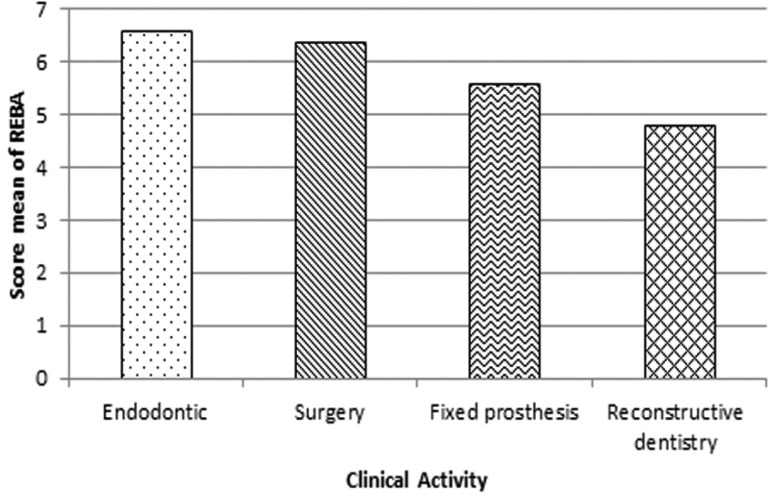
REBA mean for the students of the control and exposure groups with regard to clinical activities.

Figure 2 reveals REBA average based on clinical activities. The mean REBA score at Endodontic, surgery, fixed prosthesis and reconstructive dentistry was 6.6±2.1, 6.4±2.8, 5.6±1.7 and 4.8±2.3, respectively. The Endodontic department owns the highest REBA score, while the lowest score accounts for the reconstructive department. No significant relationship was detected between REBA scores and the type of clinical activities (*P*= 0.281), gender (*P*=0.263), duration of time spent on working with computer (*P*=0.999), experiencing musculoskeletal pain (*P*=0.158), knowledge about proper ergonomic positions (*P*=0.642), stretching between dental treatment activities (*P*=0.456) and seat adjustment before starting a clinical treatment (*P*=0.063).

**Figure 3:**
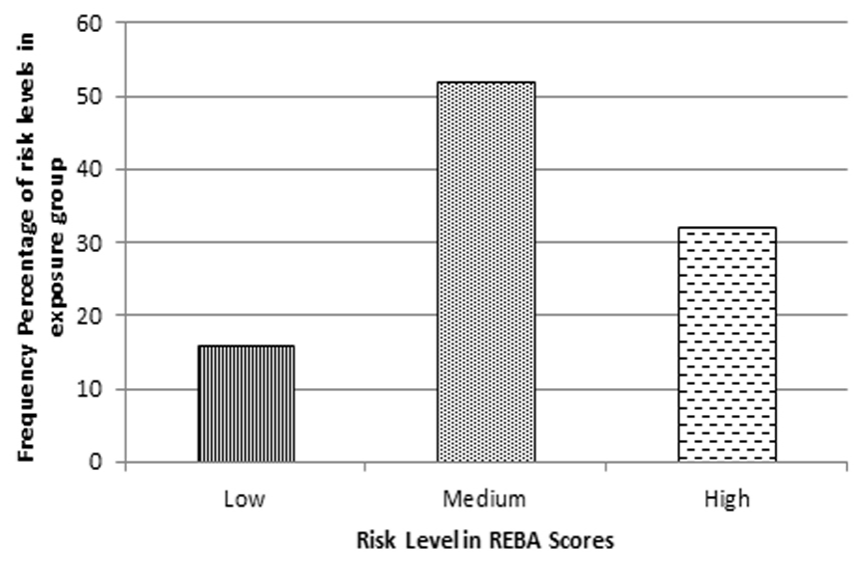
Risk level’s frequency percentage of the exposure group based on the final REBA scores.

Percentage of risk level in the exposure group is shown in figure 3. Based on the final REBA scores, 32% (19 persons) of the subjects are in the highest level of danger, 52% (31 persons) are in the medium risk level and only 16% (10 subjects) are exposed to low risk level.

## Discussion

In this study, the average musculoskeletal pains in the subjects who adjusted their seat before work was meaningfully lower than others, which is compatible with the results of the study of Caballero (5). Sim *et al*, 2006 stated the direct relation of improper postures and musculoskeletal disorders, including pain, weakness and numbness in different professions (12).

In this research, there was not any significant difference in the average pain severity in male and female students, but the average pain acuteness in the exposure group’s subjects was meaningfully more than the control group’s members. Linfors *et al.* 2006, reported that the average pain intensity in ladies is more than gentlemen (13), and in the study of Caballero *et al.* 2010 (5), the average pain intensity in female students was 83% and in male students was 41% (14). These differences with the current study might be due to the same working conditions of male and female subjects as most of the investigated dentistry students were not locals and stayed in dorms and had the same status of physical activity.

This study illustrated that students aware of ergonomic postures reported less intensity of musculoskeletal pains, but other similar researches have not paid attention to such a point. Needless to mention that, more knowledge over this field and adopting suitable work postures will be resulted in less side effects and subsequent muscular pains.

In this current study, the least average pain intensity was in reconstructive department whereas it was the most in the surgery department. Another study reported the worst working postures during extraction of the right and left lower jaw teeth with the score of 11and 10 respectively (7) which are in line with the current study. Standing postures (like the ones adopted by dentists while doing surgeries and teeth extraction) increase pain acuteness by augmenting pressure on shin and spinal cord. Hence, muscular pains are more probable in standing position than in sitting one. Based on the final results, average REBA score was the highest for those who worked in the Endodontic department and the lowest for those who worked in the reconstructive department.

One of the reasons of high final REBA scores while curing teeth roots is that, more precision is required, and the dentist is deviated from his/her normal position. Besides, high level of stress while doing endodontic treatment increases the potential risk. Teaching ergonomic principals as well as emphasizing on the usage of magnifier lens at the time of endodontic treatments could be helpful. It has been reported that, using magnifier lens optimizes dentists’ positions (14).

This study shows that doing stretching exercises after treating patients, decreases pain intensity, and obviously those who do exercises after treating patients experience less musculoskeletal pains; but this point has not been investigated in other researches.

Using computer or laptop and the hours of working with them did not have a great impact on the severity of muscular pains. Perhaps, similar activities and the same condition of the investigated students of this study made such a result. A more extended study with different dentistry groups would show preferable results.

The outcomes of this study has revealed that overall, the intensity of musculoskeletal pains in the exposure group’s students is more than the control group’s subjects. Rising *et al*, 2005 declared that the frequency and pain period in the junior students was higher than the rest (6); for sure, this is because of the fact that the junior students have already started clinical activities in diffe-rent treatment centers.

Mllis *et al*, 2004 also compared musculoskeletal pain intensity in dentistry and psychology students and stated that pain acuteness in the small of the back of dentistry students is more than others (15). It can be concluded that, ordinary people of the society do not suffer from these pains caused by improper dental work postures.

Based on final REBA records of a cross-sectional study performed among 65 dentists, Rafeemanesh *et al*, 2013 showed that 89.6% of limbs in group A and 79.3% of limbs in group B had a score more than 4 (7). In this study, similar results were obtained; 16% of the subjects were at low risk level, 52% at medium risk level and 32% at high risk level; the risk level of most students were at medium and high.

Final REBA scores demonstrated that, a noticeable population of the students was at medium (Corrective action including further assessment is necessary) and high danger levels (Corrective action including further assessment is necessary soon) ( Table 1). Reformations and sufficient ergonomic trainings are required to reduce the risk of musculoskeletal disorders in dentistry faculties.

Considering courses about profession-related musculoskeletal disorders for dentistry students, using dentistry lens for sensitive treatments and doing daily stretching exercises could help to reduce the side effects of dentistry profession.

## Conclusions

Based on the results of this study, musculoskeletal disorders are probable due to recurrent and repetitive movements of dentistry profession, prolonged working hours in static positions without sufficient breaks, incorrect work postures, implying more force and even tools and instruments; therefore, if dentistry students know about ergonomic principals of their own profession, their health is almost maintained through their activities and their lifelong.
